# Investigation of Thermal Properties of Zr-Based Metallic Glass–Polymer Composite with the Addition of Silane

**DOI:** 10.3390/polym14173548

**Published:** 2022-08-29

**Authors:** Adit Sharma, Dmitry Muratov, Mikhail Zadorozhnyy, Andrey Stepashkin, Andrey Bazlov, Artem Korol, Ruslan Sergiienko, Victor Tcherdyntsev, Vladislav Zadorozhnyy

**Affiliations:** 1 Centre of Composite Materials, National University of Science and Technology “MISiS”, LeninskyProsp 4, 119049 Moscow, Russia; 2 Scientific School “Chemistry and Technology of Polymer Materials”, Plekhanov Russian University of Economics, Stremyanny lane 36, 117997 Moscow, Russia; 3 Physico-Technological Institute of Metals and Alloys, National Academy of Sciences of Ukraine, 34/1 Vernadsky Ave., 03680 Kyiv, Ukraine

**Keywords:** metallic glass, polymer, composites, triethoxyvinylsilane, ball milling, X-ray diffraction, thermal properties, thermogravimetric analysis, fourier-transform infrared spectroscopy

## Abstract

Composites based on Zr_65_Cu_17.5_Ni_10_Al_7.5_/PTFE (polytetrafluoroethylene) with silane were prepared by ball milling with subsequent thermal pressing. Silanization was performed in the alcoholic solution with metallic glass powder. Different composites, 30/70 and 50/50 with silane, were prepared. During ball milling, Zr_2_Cu and Zr_2_Ni intermetallic phases were formed. The Zr-based metallic glass had a large supercooled region, and the melting point of the 30/70 and 50/50 composites with silane was near to the melting point of PTFE. The 50/50 composite (silane) had the highest thermal conductivity compared to the 30/70 composite samples. The incorporation of silane in metallic glass/polymer was investigated by Fourier-transform infrared spectroscopy (FTIR) and scanning electron microscope (SEM) analysis. Thermogravimetric analysis (TGA) showed the thermal stability of the composite samples up to 450–460 °C. It was also concluded that the 50/50 composite with silane has better thermal stability than the 30/70 composite with silane. The addition of silane in 30/70 and 50/50 composites increased the thermal conductivity compared to the composites without silane.

## 1. Introduction

Metallic glasses [1] have excellent mechanical properties and corrosion resistance, and are mainly produced by magnetron sputtering [2], melt spinning [3], liquid squat quenching [4], powder metallurgy [5], and various other techniques [6,7]. Metallic glasses are amorphous and donot have long-range structural order. Metallic glasses have a low Young’s modulus, high strength and hardness, and better wear and corrosion properties compared to crystalline alloys [1]. Metallic glasses are brittle and lack plasticity. Polymers have approximately low strength, low density, and high plasticity. A combination of metallic glass and polymer can show a considerate enhancement in thermal–physical properties. Metallic glass composites have improved mechanical, magnetic, thermal, and optical properties [8]. The thermodynamic conditions and kinetics of Zr-Cu-Ni-Al metallic glass were improved by the addition of Al [9]. The increase in the Ni and Al content in Zr–Cu–Ni-Al metallic glass increases the hardness, thermal stability, and mechanical properties of the composite [10]. Zr- and Cu-enriched dendrites increased the corrosion resistance in the Zr–Cu–Ni-Al coatings [11]. The enrichment of Zr in the Zr-Cu-Ni metallic glass improved the strength and plasticity under compression and exhibited a fracture strain of 3% and a yield strength of 1400 MPa [12]. Aluminum increases the thermal stability and mechanical properties of Zr-Cu-Ni-Al metallic glasses [13]. Mechanical properties, glass transition temperature, and corrosion resistance are increased by the addition of Ni in Zr-based metallic glasses [14]. The addition of Cu increases the glass-forming ability (GFA) and shows biocorrosion resistance in Zr-based metallic glasses [15]. Zr-based metallic glasses are used in biomedical instruments and in the production of nanowires [1,16].

PTFE (polytetrafluoroethylene) is used in fibers, rods, sheets, membranes/films, medical implants, and dielectric electronics [17]. The addition of PTFE to the Ni-P matrix increased its corrosion resistance. The corrosion properties were further increased when cetyltrimethyl ammonium bromide (CTAB) and polyvinylpyrrolidone (PVP) were added to the Ni-P/PTFE composite [18]. Hydroxyapatite–PTFE/Mg–Mn-Ce alloy composite has the anticorrosion properties of Mg implants in a physiological solution and is bioactive [19]. An increase in the elastic modulus of about 10.9% and a yield stress of 6.6% were predicted in the Al/PTFE composite using two-dimensional microscale finite elemental analyses (FEM) [20]. Antibacterial properties were found in the Ag-Au/PTFE composite [21]. The glass transition temperatures of the Zr_65_Cu_17.5_Ni_10_Al_7.5_ metallic glass and the melting temperature of the PTFE are very close to each other. Therefore, Zr-Cu-Ni-Al metallic glass and PTFE can be used to develop a composite for thermal properties. These properties show that PTFE can be used to prepare composites with Zr_65_Cu_17.5_Ni_10_Al_7.5_ metallic glass.

To improve the adhesion between the metallic glass and polymer, a surface modifier/ coupling agent (triethoxyvinylsilane) is used. The adhesion between Q235 carbon steel and organogel was increased by the addition of triethoxyvinylsilane. The mechanical and anticorrosion properties were also increased [22]. Zadorozhnyy et al. showed that the addition of silane increased the interaction between the metallic glass (Mg_67.5_Ca_5_Zn_27.5_) and HDPE (high-density polyethylene) [23]. The thermal conductivity and thermal resistance index were increased by the usage of silane in a glass carbon fiber-reinforced PTFE–matrix composite [24]. The effect of Zr on the Co-based catalyst showed improvement in the activity and interaction in the metal–catalyst reaction [25].

Cu_50_Zr_50_ metallic glass/polyisoprenenanolaminates showed that the composite can be used in structures and nanodevices. There was also an increase in the plasticity of the composite [26]. A uniform structure and bonding were formed in the Cu_50_Zr_45_Al_5_ metallic glass/PPS composite using microwave processing [27]. Magnetron sputtering was used to develop a nitrogen-selective TFMG/PAN composite membrane. It showed that N_2_ penetrated more than CO_2_ and O_2_, and it was concluded that the permeability of N_2_ was due to faster diffusion and pore walls [28].

In our previous studies [29], composites based on metallic glass and polymer were produced in the supercooled region (between the glass transition temperature T_g_ and crystallization temperature T_x_) by the ball milling process. In another study, a Mg_67.5_Ca_5_Zn_27.5_/high-density polyethylene (HDPE) [23] composite with triethoxyvinylsilane was produced by compression and co-extrusion techniques in the supercooled region. The Al_85_Y_8_Ni_5_Co_2_/(polyethylene terephthalate) PET [30] and (Zr_65_Cu_17.5_Ni_10_Al_7.5_/PTFE) [31] composites were prepared by ball milling in the supercooled region. In vivo studies of the Mg_66_Zn_30_Ca_4_/polycaprolactone (PCL) [32] composite was performed. These also showed that the composite was biocompatible.

The current studies continue our previous work [31]. The main criteria of our work are to produce a composite based on Zr_65_Cu_17.5_Ni_10_Al_7.5_/PTFE with triethoxyvinylsilane and have good adhesion between the metallic glass and polymer. Comparative studies of structural and thermal analysis were performed with our previous work.

## 2. Material and Methods

Induction melting was used to produce ingots of Zr_65_Cu_17.5_Ni_10_Al_7.5_ alloy (Diavac Ltd., Yachiyo, Chiba, Japan) in an argon environment by arc melting pure Zr, Cu, Ni, and Al (purity of each element used was 99.9%). Metallic glass (MG) ribbons were prepared by a single copper melting roller (under an argon atmosphere). The ribbons had a thickness of 20–30 µm. The ribbons were 2–3 mm wide. The polytetrafluoroethylene powder (PTFE-F4) had an average particle size of 10–15 µm. The samples were ball milled in an argon atmosphere using a planetary mill (Fritsch Pulverisette 5, Berlin, Germany) with a rotation speed of 300 rotations per minute (rpm). The metallic glass was ball milled for 1 h in total. The MG particles had an average size of approximately 10 µm with a measurement accuracy of about ±5 µm. We used triethoxyvinylsilane (C_8_H_18_O_3_Si- WACKER GENIOSIL^®^ GF56) to improve adhesion between the surfaces of the metallic glass and the polymer. Silanization was performed in an alcoholic solution with silane concentrations on the metallic glass ranging from 1–10 mass percent. Before applying the silane, the glass surface of the metal was cleaned with acetone and dried at 70 °C for 1 h. For the ball milling process, powdered metallic glass with silane was added to the polymer. Different composite samples were prepared with 30/70 and 50/50 metallic glass/polymer ratios (mass%). The composite samples were mixed for 1 h at a temperature of 380–400 °C with a rotation speed of 300 rpm. After mixing, the prepared powder mixture was subjected to thermal pressing in an APVM-904 hydraulic press at a pressure of 15–20 MPa and a temperature of 400 °C with the exposition of about 4 h.

Silane is used to improve the interaction between metallic glass and the polymer matrix. Silane can form an Si–O–Si bridge on organic or inorganic surfaces by cross-linking or adhesion reactions. A cross-linkage takes place due to the incorporation between the metallic glass and polymer interface [33]. The 70/30 metallic glass/polymer ratio (mass%) was difficult to prepare because of the higher content of metal and was difficult to process.

X-ray diffraction (XRD) on a DRON Diffractometer under CoKα radiation (2θ: from 10 to 100°, step: 0.2°, exposition time per step: 5 s, beam size: 6–8 mm) was used to determine the phase and structural composition of samples (Research and production enterprise “Bourevestnik”, Saint Petersburg, Russian Federation).

Thermal analysis was performed using a differential scanning calorimeter (DSC) (NETZSCH DSC 204 F1) (Netzsch Erich Netzsch GmbH & Co. Selb, Upper Franconia, Bavaria, Germany). Under an argon atmosphere, the heating rate was 10 °C/min. The sample weight ranged between 10 and 15 mg. The DSC’s maximum temperature was 600 °C for studying the supercooled region and crystallization, and 320 °C for measuring the heat capacity of the samples. A computer application was used to calculate the glass transition temperature (T_g_) and the onset of crystallization temperature (T_x_).

The thermal diffusivity of the samples was investigated using the Netzsch LFA 447 NanoFlash (Netzsch Erich Netzsch GmbH & Co., Selb, Upper Franconia, Bavaria, Germany). The temperature ranged from 25 to 300 degrees Celsius.

TESCAN-high resolution SEM, operated in a high vacuum, was used to analyze the microstructure of the composites.

Thermogravimetric studies were carried out at Thermo Scientific SDT Q600. Thermogravimetric studies were performed in the temperature range 25–650 °C with a heat rate 10 °C/min in an air flow rate 200 mL/min.

Fourier-transform infrared spectroscopy (FTIR) was performed using Thermo Nicolet 380 spectrometer with ZnSe ATR crystal in SmartiTR attachment. Every spectrum was measured in 32 scans of the mirror.

## 3. Results

### 3.1. X-ray Diffraction Analysis (XRD)

**Figure 1:**
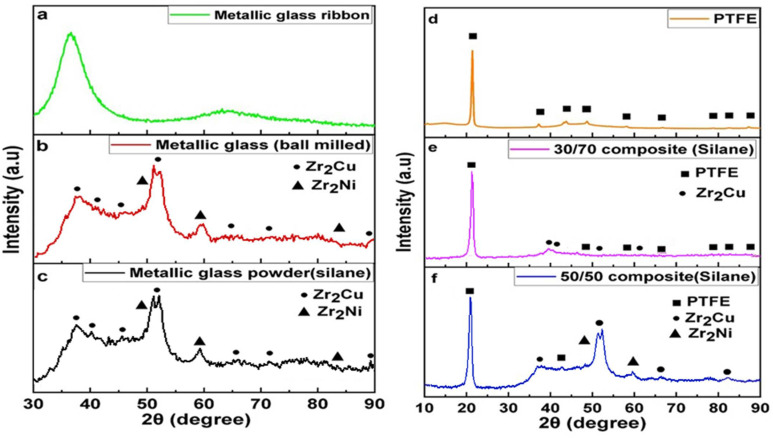
XRD pattern of the (**a**) Zr_65_Cu_17.5_Ni_10_Al_7.5_ metallic glass, (**b**) Zr_65_Cu_17.5_Ni_10_Al_7._ metallic glass after ball milling at 300 rpm, (**c**) metallic glass powder with silane, (**d**) PTFE polymer, (**e**) 30/70 composite (silane), and (**f**) 50/50 composite (silane).

X-ray diffraction shows that the metallic glass Zr_65_Cu_17.5_Ni_10_Al_7.5_ is amorphous. At 2Ɵ ≈ 30–50° in metallic glass (Figure 1a), a broad diffraction pattern can be observed. After ball milling (Figure 1b), there was a formation of Zr_2_Cu and Zr_2_Ni crystalline phases [34,35], and the metallic glass powder was semi-crystalline. There was a broad peaks pattern at 2Ɵ ≈ 31–35° and 2Ɵ ≈ 42–45° in metallic glass powder. The metallic glass powder (silane) (Figure 1c) showed similar peaks and the formation of Zr_2_Cu and Zr_2_Ni intermetallic phases compared to the ball-milled metallic glass and was semi-crystalline. The formation of most intensive crystalline peak at 2Ɵ ≈ 21° can be seen in the PTFE (Figure 1d), 30/70 (silane) (Figure 1e), and 50/50 (silane) (Figure 1f) composites. During ball milling, there was a formation of an intermetallic phase Zr_2_Cu in the 30/70 composite (silane) and Zr_2_Cu, Zr_2_Ni phases in the 50/50 composite (silane). Similar peaks were seen from 2Ɵ ≈ 30–50° in the 50/50 composite (silane) compared to the metallic glass powder after ball milling. This shows that the PTFE is crystalline; the 30/70 (silane) and 50/50 (silane) composites are semi-crystalline. In our previous results [31], metallic glass was amorphous and the formation of an intermetallic Zr_2_Cu phase was seen in the 30/70 and 50/50 composites.

### 3.2. Differential Scanning Calorimetry (DSC)

**Figure 2:**
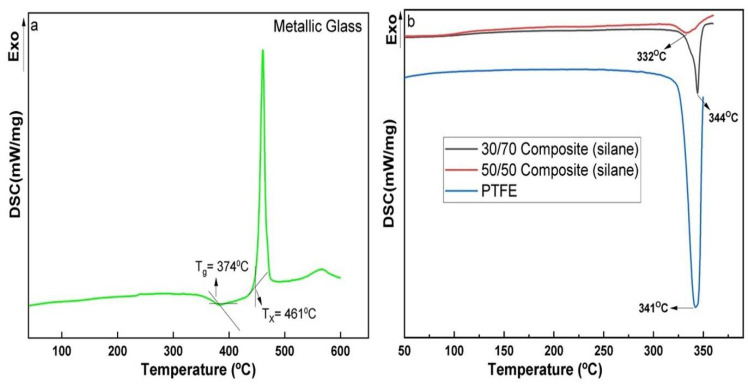
DSC analysis of (**a**) Zr_65_Cu_17.5_Ni_10_Al_7.5_ metallic glass, (**b**) PTFE, and 30/70 and 50/50 composites with silane.

According to the DSC thermogram (Figure 2), Zr_65_Cu_17.5_Ni_10_Al_7.5_ metallic glass has a T_g_ = 374 °C and T_x_ = 461 °C [31]. The metallic glass Zr_65_Cu_17.5_Ni_10_Al_7.5_ has a wide range of supercooled region of 87 °C. PTFE has a melting temperature of 341 °C. The polymer was chosen based on its physical properties and melting temperature near the supercooled region of the metallic glass. This criterion has also been used in previous works [23,29,30,31,32]. The 30/70 composite (silane) has a melting point of 344 °C, which is close to the melting point of PTFE. The melting point of the 50/50 composite (silane) sample was close to 332 °C. A similar melting point of the MG/PTFE composites, near the PTFE was also seen in our previous composite results [31].

### 3.3. Laser Flash Analysis

**Figure 3:**
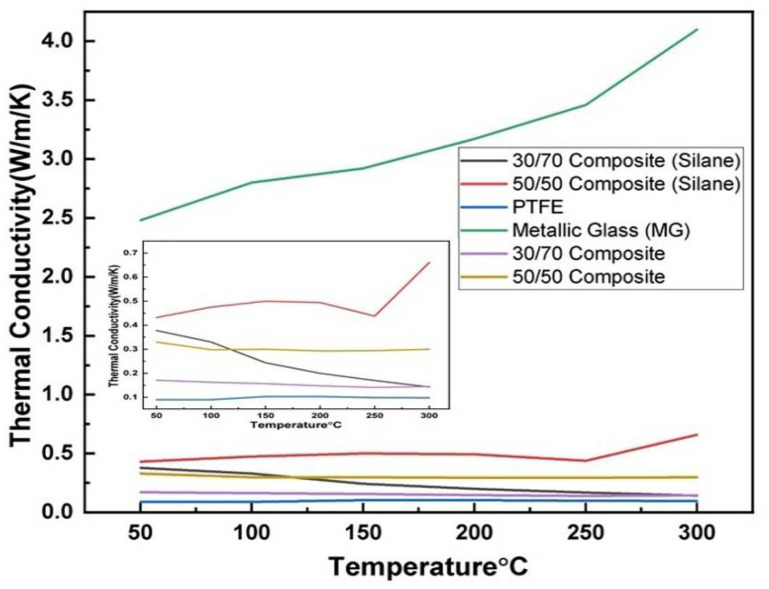
Thermal conductivity of 30/70 composite (silane), 50/50 (silane) composite, PTFE, metallic glass, 30/70 composite and 50/50 composite.

The thermal conductivity (Figure 3) of the metallic glass was highest compared to the other samples. There was an increase in the thermal conductivity (Table 1) with an increase in the temperature. The thermal conductivity of the 50/50 (silane) composite is the highest among the composites and PTFE samples. Silane improves the thermal conductivity of the 30/70 (silane) and 50/50 (silane) composites compared to our previous 30/70 and 50/50 composite samples [31]. PTFE has the least thermal conductivity compared to the samples.

### 3.4. Scanning Electron Microscope

**Figure 4:**
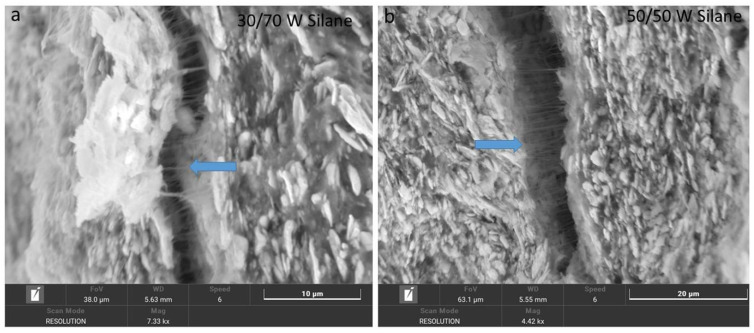
SEM images of (**a**) 30/70 composite (silane), (**b**) 50/50 composite (silane).

Silane was used to provide an adhesive interface between the metallic glass and polymer. As compared to our previous work [31], the interaction between the metallic glass and polymer has been improved. Bridges (Figure 4) (marked with arrows) can be seen in the 30/70 (silane) and 50/50 (silane) composites. This shows that there was an interaction between the metallic glass and the polymer matrix.

### 3.5. Thermogravimetric Analysis (TGA)

Thermogravimetric analysis (TGA) is used to measure the mass of a polymer/composite as a function of time or temperature in a controlled environment [36]. This allows for the evaluation of thermal stability, degradation, and other properties.

**Figure 5:**
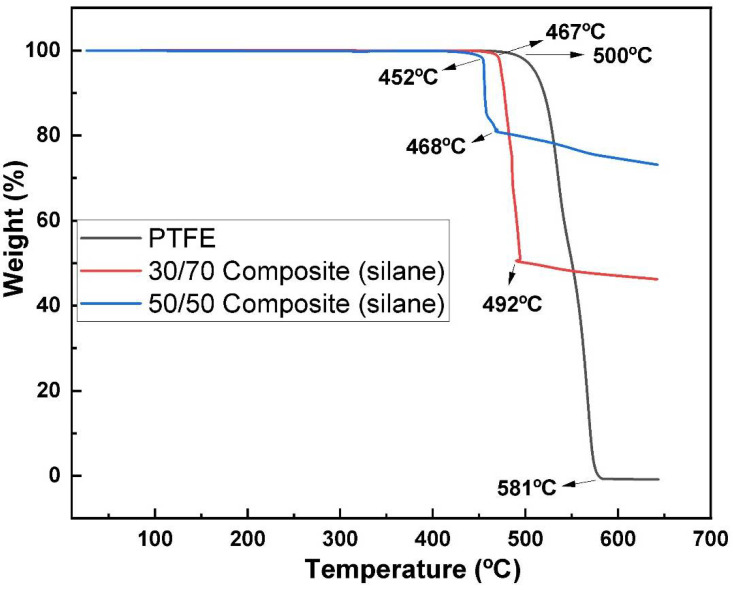
Thermogravimetric analysis (TGA) of 50/50 composite (silane), PTFE, and 30/70 composite (silane).

There was no obvious weight loss found on the differential thermogravimetric (TGA) curve (Figure 5) below 500 °C, which means it is clear that PTFE exhibited good thermal stability [37]. The 30/70 and 50/50 composites also exhibited good thermal stability in the supercooled region. At temperatures of 467 °C, 452 °C and 500 °C the decomposition of the 30/70 (silane), 50/50 (silane) composites and PTFE, respectively, was started. The weight loss percent during the decomposition of composite samples for 50/50 (silane) is 17 ± 8%, and 49 ± 5% for 30/70 (silane).

### 3.6. Fourier-Transform Infrared Spectroscopy (FTIR)

The physical constituents of polymers and their composites depend upon the arrangement of the macromolecules and their chemical constituents. FTIR spectroscopy is used to determine the conformation, tactility, and composition [38].

**Figure 6:**
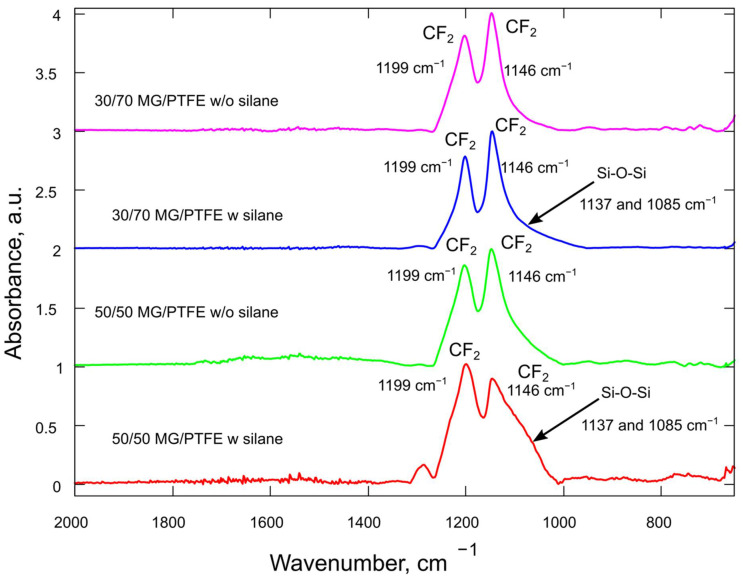
FTIR spectrum of 30/70 composite (without silane), 30/70 composite (silane), 50/50 composite (without silane), and 50/50 composite (silane).

All analyzed spectra of MG/PTFE composites (Figure 6) have two main bands of CF_2_ asymmetric stretching at 1199 and 1146 cm^−1^ [39] that are overlapped with Si-O-Si peaks. The positions of those peaks were obtained from the deconvolution of measured absorbance spectra using Gaussian functions. Two Si-O-Si bands are located at 1137 and 1085 cm^−1^ on the spectra of silane-modified composites. The sample with 50/50 MG/PTFE composition has the most intensive Si-O-Si bands (1137 and 1085 cm^−1^) that suggest the highest concentration of silane. The spectra of composites without silane do not have Si-O-Si components after deconvolution.

## 4. Discussion

The XRD pattern shows that the metallic glass is amorphous. After ball milling, the amorphous metallic glass becomes semi-crystalline and the formation of the Zr_2_Cu and Zr_2_Ni intermetallic phases occurs. The silanation process of the metallic glass has a similar pattern to that of metallic glass powder. PTFE is also semi-crystalline and shows a crystalline peak at 2Ɵ = 21°, as for the pure PTFE and so for the 30/70 (silane) and 50/50 (silane) composites. But, some kind change in the molecular chain orientation can be occurred during the ball milling process [40]. There was a formation of the Zr_2_Cu crystalline phase during the ball milling of the 30/70 (silane) composite, and Zr_2_Cu and Zr_2_Ni phases in the 50/50 composite. The formation of the broadened peaks in the XRD patterns of the metallic glass powder indicates that a nanophase is formed in the matrix [41]. In our work, nanophase Zr_2_Cu peaks were detected in a 30/70 polymer matrix similar to our previous work [31]. After the ball milling, the crystalline phase in metallic glass was about ≈ 35–40 ± 5%. The 30/70 composite (silane) has a high amount of polymer composition (70 mass %), therefore, the intensity peaks of the Zr_2_Ni phase were hidden by the background noise of the XRD pattern. This concludes that the metallic glass is amorphous, but after ball milling, the formation of Zr_2_Cu and Zr_2_Ni phases in the powder took place, therefore the final composites are semi-crystalline.

During the DSC scan, Zr_65_Cu_17.5_Ni_10_Al_7.5_ metallic glass was heated at 10 K/min. Similar to our previous results [31], metallic glass showed the onset temperature (T_g_) is 374 °C, and the onset crystallization transition temperature (T_x_) is 461 °C. The supercooled region T_sc_ = T_x_ – T_g_ of Zr_65_Cu_17.5_Ni_10_Al_7.5_ metallic glass is 87 °C. High glass-forming ability (GFA) of the Zr-based metallic glass leads to a stable supercooled region. Tao et al. discussed that the high thermal stability in the supercooled region of the Zr-based metallic glass is due to the optimization of the atomic size ratio in the densely packed random structure of the metallic glass [42]. At a heating rate of 10 K/min, PTFE, 30/70 (silane), and 50/50 (silane) composites were heated. The 30/70 (silane) composite showed a melting point near the PTFE. Similar results were obtained in our previous research [31,32]. When heat is transferred to the metallic glass during DSC heating, the composites begin to melt faster (with increasing MG content). As a result, the melting points of the composite begin to shift toward PTFE. The melting point of the polymer influences the dispersed filler in the polymer [43]. The melting point of composites could be affected by the factors listed above. Metallic glass systems are metastable and supercooling is required for glass formation. Metallic glass is metastable below the glass transition temperature (T_g_). During isothermal annealing, the metastable glass wants to reach the ideal glass state, which is known as structural relaxation [44]. Structural relaxation causes a change in the microstructure. A local rearrangement of atoms occurs for a short-range order when heat is applied to the metallic glass and results in a non-homogenous region and electron density fluctuations. Structural relaxation can be seen in 30/70 (silane) and 50/50 (silane) composites.

The thermal diffusivity of a material is defined as the amount of thermal energy a material can conduct relative to its capacity to store thermal energy. As a result, materials with higher thermal diffusivity respond more quickly to changes in their thermal environment.

The densities of the metallic glasses are lower than the crystalline solids, and they are highly alloyed solid solutions. This results in a lower value of thermal diffusivity in the metallic glass [45]. The thermal diffusivity and thermal conductivity of the metallic glass depend on the temperature and increase with an increase in the temperature. Among the samples, metallic glass has the highest thermal conductivity, whereas PTFE has the lowest. The thermal diffusivity and thermal conductivity of the PTFE decrease with an increase in temperature. PTFE is semi-crystalline and its thermal properties depend on the mean free path of the phonon. Phonon conduction dominates the heat transfer inside the material. The mean free path becomes shorter with an increase in temperature due to there being more and more interaction between the phonons. The materials become disordered during the phase change, resulting in more phonon interactions and leading to a shorter free path; hence, the thermal properties of the PTFE are decreased [46]. Another factor that changes the structure of the PTFE can be attributed to translation enthalpy. The thermal conductivity of the 30/70 and 50/50 composites was increased with the addition of silane compared to our previous results [31]. The translation enthalpy [47] alters the structure of the PTFE and causes a decrease in the thermal conductivity of the composites. The thermal conductivity of the 30/70 (silane) composite was influenced by the PTFE matrix. The thermal conductivity of the 30/70 (silane) composite was decreased due to the shorter mean free path of the phonon when the temperature was increased. The 50/50 (silane) composite has more thermal conductivity than the 30/70 composite because of its greater metallic glass content. There was a change in the thermal conductivity of the 50/50 (silane) composite at 250 °C, which can be influenced due to the incorporation of the metallic glass into the polymer matrix. After 250 °C, the increase in the thermal conductivity of the 50/50 composite was increased, similar to metallic glass. This could be due to the structural change and impact of metallic glass in a viscous polymer matrix near the supercooled region. The presence of silane bridges in 30/70 and 50/50 composites (Figure 4) increases and influences the thermal conductivity of the composite compared to the 30/70 and 50/50 composites without silane.

In our previous works [28,29,30,31], there was a lack of interaction between metallic glass and polymer. To improve the interaction between metallic glass and polymer, silane was used. The metallic glass ribbon was silanized by triethoxyvinylsilane (GF56) to improve the adhesion properties between the metallic glass and PTFE. Silanes are hybrid compounds that can act as a molecular link between organic polymers and inorganic materials [48]. By forming bridges from Si-O-Si, silane can be incorporated into either inorganic or mineral surfaces (adhesion activating or cross-linking reaction). Adhesion is activated through a cross-linking reaction made possible by silane’s ability to incorporate into the surface of the metallic glass while communicating with the polytetrafluoroethylene chain. It is also reported that the triethoxyvinylsilane can improve the thermal stability of the PTFE matrix [49].

Compared to our previous work [31], there was the formation of bridges and chemical interaction between metallic glass and the polymer matrix. This effect was observed in the main part of the contact area. Thus, it can be concluded that the addition of the silane improves the adhesion properties between the metallic glass and polymer matrix. The shrinkage of the polymer matrix during cooling is much greater than that of the metallic glass particles, and this causes the formation of the voids between the polymer matrix and the metallic glass during milling [50].

The thermal stability of the samples was measured by thermogravimetric analysis. At 500 °C, PTFE showed a single decomposition step, indicating excellent thermal stability. The 30/70 (silane) composite demonstrated good thermal stability up to 467 °C and a 47% weight loss from 467 °C to 492 °C. This can be attributed to the degradation and crystallization of metallic glass in the polymer matrix [48]. The 50/50 (silane) composite shows that the composite is thermally stable in the supercooled region compared to the 30/70 (silane) composite. There was a sudden weight loss of 17% that occurred from 452 °C to 468 °C, showing the crystallization and degradation behavior of the metallic glass in the polymer matrix. This indicates that the 50/50 composite was more thermally stable than the 30/70 composite. The addition of silanein the metallic glass and polymer matrix provides thermal stability to the composites.

The presence of the silane in the metallic glass and polymer matrix was also observed by FTIR. It was observed that the 30/70 (silane) and 50/50 (silane) composites showed that two Si-O-Si bands are located at 1137 and 1085 cm^−1^ and 50/50 (silane) has the most intensive peaks, which suggests the high concentration of the silane [51]. The composites prepared without the silane additive do not show Si-O-Si peaks.

## 5. Conclusions

Ball milling was used to produce metallic glass Zr_65_Cu_17.5_Ni_10_Al_7.5_/PTFE composites with triethoxyvinylsilane. Composites of different 30/70 (silane) and 50/50 (silane) compositions were prepared. Intermetallic phases of Zr_2_Ni and Zr_2_Cu were formed during the ball milling process. The 50/50 composite (silane) has the highest thermal conductivity compared to the 30/70 and PTFE. There was the formation of the interlinked bridges between the metallic glass and polymer matrix, showing the interaction between the metallic glass and polymer. Thermogravimetric analysis showed that the 30/70 (silane) and 50/50 (silane) composites are thermally stable and a high concentration of the silane was observed in the 50/50 (silane) composite. The decomposition in the supercooled region of the composites indicated the crystallization of the samples. FTIR analysis concluded the presence of Si-O-Si peaks inthe 30/70 (silane) and 50/50 (silane) composites. Additionally, SEM analysis showed that the interaction between the metallic glass and polymer has been improved. This suggests that silane can be used as an adhesive material between the Zr_65_Cu_17.5_Ni_10_Al_7.5_/PTFE composite and an increase in the thermal conductivity was observed in 30/70 (silane) and 50/50 (silane) compared to the 30/70 and 50/50 composites without silane.

## Figures and Tables

**Table 1:** Thermal properties of the samples.

Temperature Analysis, °C	50	100	150	200	250	300
**PTFE**						
Thermal diffusivity, mm^2^/s	0.147 ± 0.007	0.141 ± 0.001	0.134 ± 0.001	0.125 ± 0.001	0.115 ± 0.003	0.102 ± 0.007
Thermal conductivity, W·m^−1^·K^−1^	0.05 ± 0.01	0.08 ± 0.01	0.21 ± 0.01	0.29 ± 0.01	1.01 ± 0.02	0.098 ± 0.01
Heat capacity, J/(g·K)	0.421 ± 0.02	0.459 ± 0.02	0.483 ± 0.02	0.509 ± 0.02	0.540 ± 0.02	0.589 ± 0.02
Sample density, g/cm^3^	1.6 ± 0.01					
**Composite 50/50 with Silane**						
Thermal diffusivity, mm^2^/s	0.151 ± 0.03	0.148 ± 0.04	0.142 ± 0.03	0.139 ± 0.04	0.121 ± 0.03	0.114 ± 0.01
Thermal conductivity, W·m^−1^·K^−1^	0.432 ± 0.005	0.475 ± 0.007	0.500 ± 0.01	0.49 ± 0.012	0.4383 ± 0.025	0.6607 ± 0.04
Heat capacity, J/(g·K)	0.91 ± 0.03	1.02 ± 0.05	1.12 ± 0.12	1.13 ± 0.16	1.15 ± 0.22	1.84 ± 0.05
Sample density, g/cm^3^	3.15 ± 0.02					
**Composite 30/70 with Silane**						
Thermal diffusivity, mm^2^/s	0.147 ± 0.03	0.144 ± 0.03	0.136 ± 0.04	0.13 ± 0.05	0.12 ± 0.05	0.105 ± 0.05
Thermal conductivity, W·m^−1^·K^−1^	0.378 ± 0.02	0.33 ± 0.04	0.244 ± 0.05	0.20 ± 0.09	0.17 ± 0.11	0.143 ± 0.07
Heat capacity, J/(g·K)	0.96 ± 0.05	0.88 ± 0.07	0.67 ± 0.13	0.59 ± 0.21	0.55 ± 0.26	0.51 ± 0.05
Sample density, g/cm^3^	2.68 ± 0.02					
**Metallic Glass**						
Thermal diffusivity, mm^2^/s	0.466 ± 0.004	0.479 ± 0.003	0.487 ± 0.003	0.499 ± 0.002	0.505 ± 0.007	0.503 ± 0.005
Thermal conductivity, W·m^−1^·K^−1^	2.48 ± 0.1	2.80 ± 0.1	2.92 ± 0.1	3.17 ± 0.2	3.46 ± 0.2	4.10 ± 0.3
Heat capacity, J/(g·K)	1.044 ± 0.02	1.149 ± 0.02	1.177 ± 0.02	1.249 ± 0.02	1.347 ± 0.02	1.599 ± 0.02
Sample density, g/cm^3^	5.1 ± 0.01					
**30/70 Composite (without silane)**						
Thermal diffusivity, mm^2^/s	0.109 ± 0.002	0.104 ± 0.004	0.098 ± 0.007	0.09 ± 0.003	0.082 ± 0.002	0.072 ± 0.005
Thermal conductivity, W·m^−1^·K^−1^	0.171 ± 0.03	0.163 ± 0.01	0.157 ± 0.01	0.148 ± 0.02	0.141 ± 0.04	0.145 ± 0.01
Heat capacity, J/(g·K)	0.604 ± 0.02	0.605 ± 0.02	0.619 ± 0.02	0.634 ± 0.02	0.666 ± 0.02	0.775 ± 0.02
Sample density, g/cm^3^	2.6 ± 0.01					
**50/50 Composite (Without Silane)**						
Thermal diffusivity, mm^2^/s	0.178 ± 0.005	0.171 ± 0.004	0.164 ± 0.002	0.152 ± 0.002	0.144 ± 0.005	0.141 ± 0.007
Thermal conductivity, W·m^−1^·K^−1^	0.330 ± 0.02	0.298 ± 0.01	0.300 ± 0.03	0.293 ± 0.01	0.294 ± 0.01	0.3 ± 0.01
Heat capacity, J/(g·K)	0.641 ± 0.02	0.602 ± 0.02	0.631 ± 0.02	0.666 ± 0.02	6.706 ± 0.02	0.73 ± 0.02
Sample density, g/cm^3^	2.9 ± 0.01					

## Data Availability

Not applicable.
